# Being a Myeloproliferative Patient in COVID-19 Era: The Mytico Study

**DOI:** 10.3389/fonc.2021.668261

**Published:** 2021-04-15

**Authors:** Fabrizio Cavalca, Rossella Renso, Giovanni Paolo Maria Zambrotta, Carlo Gambacorti-Passerini, Elena Maria Elli

**Affiliations:** ^1^ Department of Medicine and Surgery, University of Milano-Bicocca, Monza, Italy; ^2^ Hematology Division and Bone Marrow Unit, San Gerardo Hospital, ASST Monza, Monza, Italy

**Keywords:** anxiety, myeloproliferative disease, COVID-19, burden of symptoms, quality of life

## Abstract

**Introduction:**

The Coronavirus disease 2019 (COVID-19) pandemic and the resulting social distancing, determined a reduction in access to care and limitations of individual freedom, with a consequent strong impact on quality of life (QoL), anxiety levels and medical management of onco-hematological people. In particular, in the case of patients with chronic myeloproliferative neoplasm (MPN), concern about SARS-CoV-2 infection added to the burden of symptoms (BS) which already weights on the QoL of these patients. We designed a cross-sectional survey in order to investigate the impact of the COVID-19 pandemic on status of anxiety, BS and QoL in MPN patients.

**Methods:**

We analyzed the anxiety levels using the Zung Self-Rating Anxiety Scale (SAS); BS modifications were studied using the 18 items of the Myeloproliferative Neoplasm Symptom Assessment Form [MPN-SAF].

**Results:**

132 people answered to the survey: 27 (20.4%) patients achieved a moderate to marked anxiety index value: this group described a greater worsening of symptoms than the rest of the cohort (p <0.0001). Women showed a higher level of anxiety than men (p = 0.01). A trend for lower level of anxiety was reported by patients who performed habitual physical activity (p = 0.06). A total of 98 (74.2%) patients described worsening of their symptoms during the quarantine period; 94 (71.2%) patients had postponed appointments or visits: they showed a significant worsening of their BS (p =0.01).

**Conclusion:**

This study first showed that the COVID-19 quarantine had a significant negative impact on the level of anxiety and BS in MPN patients. We identified female gender, absence of physical activity, the need for frequent visit to the hospital and the absence of a direct access to healthcare staff as the main factors associated to a higher anxiety index and worst BS.

## Introduction

In December 2019 a cluster of pneumonia cases caused by a novel coronavirus, subsequently named SARS-CoV-2, was reported in the Wuhan region of China (1). On January 31, the World Health Organization (WHO) declared the Coronavirus disease 2019 (COVID-19) a public health emergency of international concern due to the burden on the healthcare system and lack of specific treatments (2).

The first indigenous case of SARS-CoV-2 infection was diagnosed in Italy on February 21, 2020. National restrictions varied from total lockdown to targeted quarantine and social distancing (3). Despite drastic efforts, the World Health Organization (WHO) officially declared the COVID-19 pandemic on March 11, 2020 (4). The COVID-19 pandemic and the resulting social distancing and lockdown measures determined a substantial impact on daily life and medical management of cancer patients, including hematological people (5). The rapid and uncontrolled spread of the virus forced Italian hospitals to undergo an emergency reorganization to prioritize medical resources for COVID-19 patients: as a consequence, for many patients, including hematological ones, exams and visits have been postponed to a later date (6).

In the field of hematological diseases, we focused on chronic myeloproliferative neoplasm (MPN), including polycythemia vera (PV) essential thrombocythemia (ET) and myelofibrosis (MF). These are clonal disorders characterized by uncontrolled proliferation of one or more cell type of the myeloid lineage (7), responsible of a high symptomatic burden (BS) which affects quality of life (QoL) of these patients (8); this include a high prevalence of constitutional or microvascular symptoms (i.e. itching, sweating, weight loss, fatigue, early satiety, anorexia and fever) as well as a reduction in the emotional wellbeing and the ability to work (9). QoL improvement represents today a fundamental therapeutic endpoint for the management of MPN patients. Mesa et al. first proved that BS of MPN patients negatively affects QoL, daily living, and the ability to work (10).

Delays and changes in hematological treatment protocols due to COVID-19 may induce concerns about recurrence, progression of disease or survival in hematological patients. This, in associations with the new concerns related to the pandemic, could impair patients’ mental and emotional wellbeing and create a state of anxiety (11). Measures of social distancing or lockdown may interfere with networks of support, having a negative impact on mental health and emotional functioning. Moreover, a high proportion of patients on active treatment for MPN are constantly exposed to medical facilities and healthcare staff, which puts them at higher exposure risk. Fear of infection, related to frequent hospital transits and visits for outpatients therapies, laboratory checks or transfusions in hospital, could have conditioned MPN patients, who have often preferred to postpone their visits and check-ups, or relying on an electronic control (via phone calls or e-mail).

We designed a cross-sectional survey, the MYTICO study (Being a Myeloproliferative Patient in COVID-19 era) in order to investigate the impact of the COVID-19 pandemic on status of anxiety, BS and QoL in MPN patients.

## Material and Methods

This is a cross-sectional study conducted to investigate prevalence, severity and factors related to anxiety in MPN patients during COVID-19 pandemic. We also evaluated changes in BS of disease during lockdown for COVID-19. Data were collected in the period from 25th May to 30th June 2020, at the end of first COVID-19 lockdown in Italy.

Study participants included all patients ≥18 years old, with diagnosis of Ph negative MPN (PV, ET, MF) according to WHO 2016 criteria (12), who are followed at Myeloproliferative Disease Center of the Hematological Division of the S. Gerardo Hospital in Monza (Italy).

### Ethics

Ethical approval was not required because this study did not involve a prospective evaluation.

Participation in this study was completely voluntary. The questionnaire started with informed consent. The patients/participants provided their informed consent before proceeding with the subsequent investigation.

This study was in accordance with the ethical standards of the institutional and/or national research committee and with the 1964 Helsinki declaration and its later amendments or comparable ethical standards.

### Data Collection

The survey was conducted by administering an online multi-questionnaire with closed-ended questions, divided into four sections, in order to collect information regarding:

(1) Demographic characteristics, such as gender, age, Body Mass index (BMI), educational level (no education, primary school, secondary education, higher professional education, or university degree), type of occupation, habitual physical activity;
(2) MPN-related features: type of MPN (PV, ET, MF), year of diagnosis, type of therapy and how to access to treatment (including difficulty in finding drugs during the lockdown);
(3) Socio-relational and occupational aspects: type of occupation, habitual physical activity, history of direct contact with people with Sars-CoV-2 infection, or died by Sars-CoV-2 infection or if they themselves had received a diagnosis of SARS-CoV-2 infection; quality of communication with hematologist (modification in relationship with the health staff – family doctor; hematologist- during lockdown);
(4) Measurement of anxiety levels: Zung Self-rating Anxiety Scale (SAS) was used to assess anxiety levels in MPN patients (13). It is a 20 items self-report assessment tool designed to measure anxiety levels. Each question is scored on a 1–4 scale, with total raw scores ranging from 20 to 80. The SAS scores were divided into four categories: normal range, (⩽49), mild to moderate anxiety (50–59), marked (60–74) and severe anxiety (⩾75), after standardizing the score based on raw data multiplied by 1.25. Previous studies have shown that SAS internal consistency reliability was 0.66–0.80 and the Cronbach’s *α* was 0.87 (14);
(5) Measurement of symptom burden of disease: Myeloproliferative Neoplasm Symptom Assessment Form [MPN-SAF] was used to evaluate the symptoms MPN-related (15). MPN-SAF is an 18-item instrument designed to assess prevalence and severity of symptoms of MF, PV and ET, and has been validated in international trials. Based on the 18 items of the MPN-SAF we investigated the modification of symptoms related to myeloproliferative disease during the lockdown period: in particular, we analyze if the symptoms remained identical, improved slightly, improved greatly, slightly worse or much worse. If the symptoms remained identical, a score of 0 points was assigned; if they improved slightly, score 1; if they greatly improved, score 2; if they worsened slightly, score -1; if they got a lot worse, score -2.

The survey was conducted among all MPN participants with a known and active email address, who voluntarily replied to each section of the multi questionnaire.

### Statistical Analysis

For this analysis, no *a priori* statistical calculation of the sample size was implemented, also considered the absence of similar reference data in the literature. Numbers and percentages are used to describe categorical variables. Continuous and categorical variates were summarized as median values ± standard deviation (s.d.) and frequency (percentage), respectively. We used the *χ* ^2^ test to identify the differences in categorical variables between groups, and the Student’s *t* test was used to determine the differences in continuous variables between groups. If the data showed a skewed distribution, the Mann–Whitney test was used. In addition, a univariate analysis model was used to identify the relationship between risk factors and anxiety score. Finally, our paper also lists the unadjusted and adjusted multivariate linear regression analysis model.

Statistically significant differences were identified as a two-sided *P* value <0.05.

## Results

On 220 patients who received the online survey, 132 (60%) patients replied; of them 74 were women (56%) and 58 men (44%). The median age of participants was 62.5 (range: 26-95) years. According to diagnosis, 37 (28%) patients were affected by MF, 29 (22%) by PV and 66 (50%) by ET. Median time from diagnosis of MPN was 8 (range: 0-32) years, respectively 8 for MF (range: 1-32), 6 for PV (range 1-27) and 8 years (range: 0-30) for ET patients. The median visit frequency was 3 months (range 1-12).

Two patients were diagnosed with SARS-CoV-2 infection (1.5%). 11 patients (8.3%) reported having had contact with people tested positive for SARS-CoV-2 infection; 5 of them, had a close contact (family or friend) who died for SARS-CoV-2 infection (3.8%).

About the ongoing therapeutic program, 107 (81%) patients received an active treatment including cytoreduction and/or phlebotomy; in 102 (77%) of them, at least one cytoreductive drug was administered. **Table S1** show the details of the different treatments.

Only 6 patients (4.5%) reported difficulty in finding the habitual therapy during lockdown.

Regarding their educational level, 25 (18.9%) have achieved university degree; 58 (43.9%) graduated from high school, 24 (18.1%) middle school, 25 (18.9%) elementary school.

The average BMI was 25.5 (median 24.83 - range: 17.5 – 41.8), without differences for MPN type. Ninety patients (68.2%) reported having contacted the hematologist in a different way than usual during the quarantine period (e-mails, telephone visits).

Only 37 (28%) people continued to work during the quarantine period and 66 (50%) patients reported that they normally perform habitual physical activity.

Overall, 72 (54.5%) patients reported that their QoL has globally reduced during the lockdown (**Table S2**).

### Measurement of Anxiety Levels

The mean anxiety index was 42 (range: 26-66), which was classified as normal according to the SAS. Overall, 104 (78.8%) patients had a level of anxiety describable as normal, 22 (16.6%) as mild to moderate; 5 (3.8%) as marked.

Women showed a higher level of anxiety than men (43 vs 39; p=0.01). The group of patients (50%) who reported to perform the habitual physical activity, presented a trend regarding lower level of anxiety compared to those who usually do not perform physical activity (43 vs 39, p = 0.06). We found no difference in the level of anxiety for those who worked during the quarantine period or not, or for BMI, or for educational level.

The group of patients who had close contact (family or friend) who died for SARS-CoV-2 infection (3.8%) showed a trend regarding a higher level of anxiety (48 vs 40; p=0.07). Otherwise, no differences were recorded for those who had contact with patients affected by SARS-CoV-2.

No significant differences were showed about the anxiety’s level based on the type of MPN and other demographic and socio-occupational features (**Table 1**).

**Table 1:** Principal variables and correlation with SAS index score and burden of symptoms (BS).

Variables	N (132)	Column A		Column B	
	n, (%)	SAS index score (Anxiety)	P	MPN SAF modification (Symptoms)	P
**Sex**			**0.0145**		0.4802
M	58 (44%)	39		-2.5	
F	74 (56%)	43		-3	
**Age (years)**			0.9237		0.3016
<60	54 (41%)	41		-3	
>60	78 (59%)	40.5		-2	
**Type of MPN**			0.07881		0.4124
MF	38 (29%)	42		-3.1	
PV	29 (22%)	43		-3.8	
TE	65 (49%)	42		-2.7	
**Close contact died due to Covid-19**			0.0754		0.3632
Yes	127 (96%)	40		-5	
No	5 (4%)	48		-2	
**Close contact affected by to Covid-19**			0.516		0.6142
Yes	11 (8%)	43		-3	
No	121 (92%)	41		-3	
**Received a diagnosis of Covid-19**			0.1209		0.9255
Yes	2 (2%)	52		-2.5	
No	130 (98%)	41		-3	
**Frequency of visits**			0.2437		**0.0275**
<1 month	17 (13%)	45		-6	
>1 month	115 (87%)	40		-2	
**Habitual physical activity**			0.0602		0.2832
Yes	66 (50%)	39		-2	
No	66 (50%)	43		-3	
**Anxiety level**					**<0.0001**
Normal*	105 (80%)	39		-2	
More than normal	27 (20%)	55		-9	
**Cytoreduction (No/Yes)**			0.9946		0.6870
Yes	102 (77%)	40,5		-3	
No	30 (23%)	41		-2	
**Difficulty in drug's supply**			0.6481		0.746
Yes	126 (95%)	41		-3	
No	6 (5%)	42.5		-3,5	
**Appointment postponed**			0.1345		**0.0158**
Yes	94 (71%)	41		-3	
No	38 (29%)	38.5		-1,5	
**Time from diagnosis (years)**			0.5696		0.3938
<2	23 (17%)	40		-2	
>2	109 (83%)	41		-3	
**BMI >25**			0.0964		0.4607
>25	68 (52%)	44		-2	
<25	64 (48%)	40		-3	
**Worked during the lockdown**			0.1301		0.6456
Not worked	95 (72%)	41		-3	
Worked	37 (28%)	40		-3	
**Weight gain during the lockdown**			0.3133		0.9366
Yes	56 (42%)	39.5		-3	
No	76 (58%)	43		-2,5	
**Relationship with the haematologist on a scale from 1 to 5**			0.517		**0.0324**
>4	127 (96%)	41		-10	
<4	5 (4%)	46		-2	

M, male, F, female; MPN, chronic myeloproliferative disease; MF, myelofibrosis; PV, Polycythemia Vera; ET, Essential Thrombocythemia; BMI, Body mass index.

* a "normal" range of anxiety is defined by a score <49 on the Zung self-rating anxiety scale. Bolded text represent statistically significant p values.

### Measurement of BS

98 (74.2%) patients described a worsening of their burden of MPN related symptoms during the quarantine period; for another 17 people BS remained the same (12.9%), while another 17 (12.9%) described a general improvement. Twenty-seven (20.4%) patients achieved a moderate to marked anxiety index: this group presented a significant worsening of symptoms than the rest of the population (-9 vs -2; p <0.0001).

According to modality and frequency to access in Hospital, patients reported visiting every 3.5 (range: 0-6) months. Patients who had monthly visits (n=17, 12.9%) reported greater worsening of their BS than those who visit less frequently (-6 vs –2; p =0.02). Therefore, people who had postponed their appointments or visits (n=94, 71.2%) showed a significant worsening of their BS (-3 vs –1.5; p=0.01). These patients had no significant different features compared to the rest of people of the survey.

On a scale of 1 to 5, patients rated their relationship with the hematologist with an average score of 4.78. The group of patients (n=5, 3,8%) who rated the relationship with the hematologist with a score <4 (on a scale from 1 to 5), have shown during the lockdown a significant worsening of BS (p =0.03)

No significant differences were showed about the modification of BS based on the type of MPN and other demographic and socio-occupational features (**Table 1**).

## Discussion

MPN are clonal pathologies encumbered by an important BS. Despite the different profiles of each MPN, all seem to be burdened by the same symptom spectrum. The 2017 edition of the National Comprehensive Cancer Network (NCCN) guidelines has, for the first time, included symptomatic assessment into the response criteria for MPN (16). The lockdown established by the Italian government had a strong psychological impact on the population and, in particular, on hematological patients.

Among patients with a Sars-CoV-2 infection, those affected by hematological disease have been shown to have a higher mortality (17). In fact, compared with the general Italian population with COVID-19, the standardized mortality ratio of hematological patients with COVID-19 was 2·04 (95% CI 1·77–2·34) and 3·72 (2·86–4·64) in individuals younger than 70 years. Moreover, Passamonti et al. (18) recently reported in a multicenter study that 32.5% of patients with MPN, who contracted SARS-CoV-2 infection, died. Anxiety status given by the SARS-CoV-2 pandemic has therefore added to the concern given by the hematological pathology, impacting strongly on QoL of patients with MPN.

This study first showed that COVID-19 quarantine had a negative impact on the level of anxiety and BS in MPN patients: about half (54.5%) of patients reported that their QoL has globally reduced during the lockdown.

Regarding the assessment of anxiety levels, we showed that the group of patients which reported a moderate to marked anxiety index (20.5%) presented a significantly worsening of symptoms than those with a level of anxiety classified as “normal” (p<0.0001). A poor ability to recognize a state of anxiety has been described as being linked to a lower survival in cancer patients (19). A greater level of anxiety correlates directly with a worsening of MPN-related symptoms: so, it appears clear that anxiety created by the threat of infection from COVID-19 may directly influence the BS already existing due to the hematological disease. This is also demonstrated by the fact that 37 patients (28%) reported that the burden of concern for hematological disease worsened during the quarantine, while only 6 (4.5%) stated that they found an improvement in symptoms in the same period.

Our data on impact of COVID-19 pandemic on the anxiety’s level are similar to those reported by Romito et al. (20) in the setting of lymphoproliferative diseases. In this study the authors analyzed the psychological status of outpatients receiving infusion and not deferrable chemo- or immunotherapy for lymphoproliferative neoplasms. They observed that 36% presented with anxiety (HADS-A), 31% depression (HADS-D), and 43% were above the cut-off for the HADS-General Scale. Furthermore, in our study we found that female patients presented a higher anxiety level than male (p<0.01). This data is consistent with what reported in the literature by Rossi et al. (21); however, in contrast with Geyer et al. (9), we found no gender differences in our experience regarding BS modification during the quarantine period. Besides, according to the literature, patients who habitually perform physical activity were shown to have a trend for lower level of anxiety (p = 0.06) (22).

Focusing on BS, almost 75% of patients reported a worsening of their symptoms during lockdown. We found that patients who reported going to the hospital at least once a month reported a greater worsening of BS than other group: this may be due to more severe illness or worsened more significantly during the period of the lockdown, and not necessarily be a direct consequence of it. On the other hand, these patients were also those on active therapy, and therefore, more subjected to the danger of incurring the contagion of COVID-19 by going to the hospital.

Lastly, the reorganization of healthcare facilities during the COVID-19 pandemic has given a new role to telemedicine (23). In a recent survey of Gruppo Italiano Malattie Ematologiche dell’Adulto (GIMEMA) most Italian hematologists declared to have temporarily adopted telephone or video consultations in patients with MPNs during COVID-19 pandemic in order to reduce the inflow of patients and the risk of contagion at each hematological center. They also shared a certain propensity to expand the use of telemedicine after pandemic resolution. Compared to patients receiving a telephone contact, the patients who required in person visits were more frequently affected by MF, under active therapy and enrolled into a clinical trial. Although telemedicine resulted in an overall good level of patients’ satisfaction, the lack of physical interaction was perceived as a strong limitation by all patients, emphasizing how much hematological care moves beyond the technical evaluation of blood tests, and instead involves a global patient–doctor relationship (24, 25). Even in our study, 68.2% of patients (n=90) reported to have communicated with the caregiver with new means compared to the usual ones. Although there were no differences with respect to the BS and the level of anxiety between people who used telemedicine and those who did not, it must be remembered that the systematic and exclusive use of these modalities could undermine the clinical sensitivity and the feeling with the hematologist and be limiting in the care of the subgroup of more fragile people and with less feeling with technology. Remote consulting might also be less suited to vulnerable patients and individuals from low socioeconomic backgrounds than to patients from high socioeconomic settings (26). The importance of proximity with medical staff is demonstrated by the fact that those who had postponed appointments had a worsening of BS; similarly, patients who positively assessed the relationship with the medical staff had a minor worsening of BS during the quarantine period. All these aspects underline that proximity with medical staff is essential to correctly inform and reassure patients about their disorders and to ensure optimal treatment especially for those patients who are taking immunotherapy such as Ruxolitinib or Interferon. Furthermore, COVID-19 cases are rising daily around the globe, placing the clinician in front of still open questions regarding the optimal management of these patients (thrombotic risk, management of cytoreductive therapy during infection, etc.) (27).

There are some limitations to this work: firstly, the monocentric nature of our study, conducted in a center of northern Italy, one of the areas most affected by the pandemic. Second, even though baseline characteristics of responders and non-responders should be comparable, an under- or overestimation of the results due to selective (non-)response could not be ruled out as the reasons for 40% non-response were unknown. Similar survey in oncological setting were reported in literature in studies conducted with the same modality of administration (28).

Moreover, we do not have a baseline (pre-COVID-19) evaluation regarding the levels of anxiety and BS of MPN patients who answered at the survey, in order to quantify changes in anxiety severity index and BS measurements in a more objective way. Furthermore, the addition to the online questionnaire of a specific score for the QoL, such as EORTC-QLQ-C30, would have allowed us to take a more in-depth look at this aspect as well (29). At last, our study measured the impact of COVID-19 approximately four weeks after the start of COVID-19 pandemic. Therefore, it is unclear whether the results of this study represent a short-term, or a longer-lasting effect. However, previous literature on the 2009 H1N1 viral threat showed that the psychological effects can persist up to 30 months after the outbreak (30).

## Conclusion

This study first showed the negative impact of COVID-19 pandemic on the level of anxiety and BS in MPN patients. We identified female gender, absence of physical activity, the need for frequent visit to the hospital and the absence of a direct line with the healthcare staff as the main factors associated to a higher anxiety index and worst BS. However, further studies with a longer follow-up and a larger sample size are needed, in order to provide adequate interpretations and support measures to improve the psychological well-being of patients and help to optimize the local reorganization of healthcare facilities, focusing on the relationship between medical staff and patients.

## Data Availability Statement

The raw data supporting the conclusions of this article will be made available by the authors, without undue reservation.

## Ethics Statement

Ethical approval was not required because this study did not involve a prospective evaluation. Participation in this study was completely voluntary. The questionnaire started with informed consent. The patients/participants provided their informed consent before proceeding with the subsequent investigation. This study was in accordance with the ethical standards of the institutional and/or national research committee and with the 1964 Helsinki declaration and its later amendments or comparable ethical standards.

## Author Contributions

Each author has contributed significantly to, and is willing to take public responsibility for, the following aspects of the study: Design: FC, EE. Data collection: FC, GZ, RR. Analysis and Interpretation: FC, EE. Drafting: FC, EE. Critical revision: FC, EE, RR, GM, CG-P. All authors contributed to the article and approved the submitted version.

## Conflict of Interest

The authors declare that the research was conducted in the absence of any commercial or financial relationships that could be construed as a potential conflict of interest.
