# Supplementary material for: Being a Myeloproliferative Patient in COVID-19 Era: The Mytico Study
Source: Front Oncol. 2021 Apr 15;11:668261. doi: 10.3389/fonc.2021.668261 (PMC8082139; doi:10.3389/fonc.2021.668261)
Supplement: Supplementary file 1 [file Table_1.pdf]

Active treatment (cytoreduction or phlebotomy)

Yes	107 (81%)
No	25 (19%)

Cytoreduction

Yes	102 (77%)
No	30 (23%)

Type of cytoreduction

Hydroxyurea only	*75 (73,53%)*
Hydroxyurea and Anagrelide	*3 (2,94%)*
Hydroxyurea and Ruxolitinib	*1 (0,98%)*
Hydroxyurea and Busulfan	*1 (0,98%)*
Hydroxyurea and Interferon	*4 (3,92%)*
Anagrelide	*3 (2,94%)*
Ruxolitinib	*10 (9,8%)*
Busulfan	*4 (3,92%)*
Anagrelide and 6-mercaptopurine	*1 (0,98%)*

Phlebotomy

Yes	10 (8%)
No	122 (92%)

Table S1: Characteristics of patients according to the treatment administered.
