# Supplementary material for: Being a Myeloproliferative Patient in COVID-19 Era: The Mytico Study
Source: Front Oncol. 2021 Apr 15;11:668261. doi: 10.3389/fonc.2021.668261 (PMC8082139; doi:10.3389/fonc.2021.668261)
Supplement: Supplementary file 2 [file Table_2.pdf]

Categories	N (%)
**Quality of life**	
Greatly improved (1)	6 (4,5%)
Slightly improved (2)	7 (5,3%)
Identical (3)	46 (34,8%)
Slightly worsened (4)	58 (43,9%)
Greatly worsened (5)	14 (10,6%)

Table S2: Overall change in quality of life during the lockdown period.
